# Cardioprotective activity of flax lignan concentrate extracted from seeds of *Linum usitatissimum* in isoprenalin induced myocardial necrosis in rats

**DOI:** 10.2478/v10102-011-0016-8

**Published:** 2011-06

**Authors:** Anand A. Zanwar, Mahabaleshwar V. Hegde, Subhash L. Bodhankar

**Affiliations:** 1 Department of Pharmacology, Poona College of Pharmacy, Bharati Vidyapeeth Deemed University, Paud Road, Erandwane, Pune-411 038, Maharashtra, India; 2 Interactive Research School for Health Affairs, Medical college campus, Bharati Vidyapeeth Deemed University, Dhankawadi, Pune-411 043, India

**Keywords:** flax lignan concentrate, flaxseed, isoprenalin, myocardial necrosis, *Linum usitatissimum*

## Abstract

The objective of the study was to evaluate the cardioprotective activity of flax lignan concentrate (FLC) in isoprenalin (ISO) induced cardiotoxicity in rats. Male Wistar rats (200–230 g) were divided into three groups. Group I: control, Group II: isoprenalin, Group III: FLC (500 mg/kg, p.o.) orally for 8 days and in group II and III isoprenalin 5.25 mg/kg, s.c. on day 9 and 8.5 mg/kg on day 10. On day 10 estimation of marker enzymes in serum and haemodynamic parameters were recorded. Animals were sacrificed, histology of heart was performed. Isoprenalin showed cardiotoxicity, manifested by increased levels of marker enzymes and increased heart rate. FLC treatment reversed these biochemical changes significantly compared with ISO group. The cardiotoxic effect of isoprenalin was less in FLC pretreated animals, which was confirmed in histopathological alterations. Haemodynamic, biochemical alteration and histopathological results suggest a cardioprotective protective effect of FLC in isoprenalin induced cardiotoxicity.

## Introduction

Ischaemic heart disease is a leading cause of morbidity and mortality worldwide. The pathophysiology of cellular damage due to ischaemia is complex. Reactive oxygen species are known to play a major role and are a target for therapeutic interventions (Li & Jackson, 2002). It is well recognized that ischaemic tissue generates oxygen-derived free radicals and other reactive species which bring about oxidative damage of membrane lipids, proteins and carbohydrates leading to qualitative and quantitative alterations of the myocardium (Burton *et al*., 1984). Isoprenalin (ISO), a synthetic catecholamine and β-adrenergic agonist is documented to produce myocardial infarction due to generation of highly cytotoxic free radicals through its auto-oxidation (Rona *et al*., 1959). Higher levels of catecholamines deplete the energy reserve of cardiac muscle cells, leading to complex biochemical and structural changes that cause irreversible cellular damage and ultimately necrosis (Rona, 1985).

Isoprenalin-induced myocardial necrosis in animals [either by subcutaneous (s.c.) or intraperitoneal injection] was first reported by Rona *et al*. (1959). The biochemical and histological changes occurring after administration of this agent in rats have been well documented. The pharmacologic effect of isoprenalin is believed to be associated with its β-adrenergic effect, which increases heart rate, decreases blood pressure and diminishes the oxygen supply to the myocardium. Within 6 min of intraperitoneal isoprenalin injection, histological changes occur, including myofilament fragmentation (Kung & Blau, 1978).

*Linum usitatissimum* (Linn.), commonly known as flaxseed or linseed belongs to the family Linaceae. The flax plant is not a new crop and is native to West Asia and the Mediterranean. As the source of linen fiber, flax has been cultivated since at least 5000 BC (Oomah, 2001). Traditionally, flaxseed has been grown for its oil. Flaxseed has been playing a major role in the field of diet and disease research due to its potential health benefits associated with high content of α-linolenic acid (ALA) (57%), which is an essential omega-3-fatty acid and also because of a major lignan, namely secoisolariciresinol diglucoside (SDG). Flaxseed is an oilseed that contains about 38–45% oil. ALA content in flax oil is 55–60% and lignan content in flaxseed is up to 13 mg/g flaxseed (Hall *et al*., 2006). The lignan SDG is converted by bacteria in the colon of humans and other animals to enterodiol and enterolactone, referred to as mammalian lignans. These exhibit weak estrogenic activity as they can bind to estrogen receptors on cell membranes (Nesbitt *et al*., 1999). The interest in ALA and lignans has opened opportunities. Flaxseed is also an good source of protein and dietary fibre, accounting for 20% and 28% of the flaxseed, respectively (Hall *et al*., 2006).

With better understanding of the process involved in the pathophysiology of myocardial infarction, it is possible to investigate the cardioprotective ingredient in food. Many dietary antioxidants and some non-nutrient based antioxidants from plants, such as sulphur containing compounds in garlic, phyto-oestrogens in soy, green tea, anthocyanins in red berries, lycopene in tomatoes, red and white wines from grape seeds are increasingly being recognized as potential health promoters in reducing the risk of cardiovascular disease and atherosclerosis (Ames *et al*., 1993). Flaxseed has gained much importance in recent times as ethnomedicine due to its wide pharmacological actions. Although its therapeutic potential, as antioxidant, primarily as hydroxyl radical scavenger, anticancer, antidiabetic antiviral, bactericidal, anti-inflammatory, and antiatherosclerotic agent is known (Zanwar *et al*., 2010; Rajesha *et al*., 2006; Chen *et al*., 2002; Prasad, 2000; Collins *et al*., 2003; Kinniry *et al*., 2006; Prasad, 1997), very few studies evaluating its cardioprotective potential are presently available (Penumathsa *et al*., 2008, 2007). The objective of the present investigation was to study the effect of flax lignan concentrate (FLC) extracted from seeds of *Linum usitatissimum* on isoprenalin induced cardiotoxicity in Wistar rats.

## Materials and methods

### Collection and authentication of plant

Authenticated seeds of *Linum usitatissimum* were obtained from Dr. P. B. Ghorpade, Principal, Scientist and Linseed Breeder, Punjabrao Deshmukh Krushi Vidyapeeth, College of Agriculture, Nagpur, India, Maharashtra State, India, and voucher specimen was deposited at the institute.

### Drugs and chemicals

Isoprenalin (ISO) (Sigma Chemicals, St. Louis, USA) was procured for induction of myocardial necrosis. Absolute alcohol (Changshu Yangyuan Chemicals, China) was purchased from respective vendors. n-Hexane, hydrochloric acid, sodium hydroxide and sodium chloride of analytical grade were purchased from Qualigene fine-chem. Ltd, Mumbai, India. Solvents (ethyl acetate, methanol, formic acid) and precoated silica gel plates of 0.2 mm. thickness (silica gel 60 F-254) used for the present study were procured from Merck Ltd. Mumbai, India.

### Preparation of flax lignan concentrate (FLC)

Several methods have been reported for extraction of lignan (Charlet *et al*., 2002; Eliasson *et al*., 2003; Muir & Westcott, 2000; Meagher *et al*., 1999; Fritsche *et al*., 2002). We prepared lignan extract by using these methods and analysed the SDG content. The highest SDG content could be extracted by the method of Eliasson *et al*. (2003). Briefly, the double cold pressed flaxseed cake/meal (200 g) was obtained from the Indian Council of Agricultural Research under National Agriculture Innovation Project, Omega-3-oil unit, Sangamner, Maharashtra, India. This seed cake was defatted by n-hexane in soxhlet apparatus to remove residual oil. The defatted cake was then hydrolysed with 1 M aqueous sodium hydroxide for 1 h at room temperature with intermittent shaking, followed by extraction with 50% ethanol. Then the filtrate was acidified to pH 3 using 1 M hydrochloric acid. The filtrate was dried on a tray dryer at 50 °C. The yield of FLC was 14.81% w/w. The powdered ethanolic extract was dissolved in distilled water to prepare different concentrations of FLC and used for chromatographic analysis and pharmacological studies.

### Preparation of standard solutions and FLC for chromatographic analysis

Stock SDG standard solution (concentration 2 mg/ml) was prepared by dissolving 2 mg of pure SDG in 1 mL of double distilled water assembly (Labponko, Missoruri, USA). The working solution was prepared by further dilution in methanol to make final concentration as 2, 4, 6, 8, 10, 12 and 14 µL of these were applied to a thin layer chromatography (TLC) plate, using an automatic TLC sampler (Linomat 5) for preparing six point calibration curves. FLC was dissolved in distilled water and further appropriate dilutions were made in methanol.

### Chromatography

A Camag high-pressure thin-layer chromatography (HPTLC) system equipped with an automatic TLC sampler (Linomat 5), TLC scanner 3, and integrated software Win-Cats version 4 was used for the analysis. HPTLC was performed on a precoated silica gel HPTLC 60F254 (20×10 cm) for the quantification of SDG in FLC. The samples and the standards were applied on the plate as 8 mm wide bands. The linear ascending development was carried out in a camag twin trough chamber (20×20 cm), which was pre-saturated with 30 mL mobile phase with ethyl acetate: methanol: water: formic acid (7:1.5:1:0.5) for 15–20 min, at room temperature. The length of the chromatogram run was up to 80 mm. Subsequent to the development, the TLC plate was dried in a current of air and with a hair dryer. Quantitative evaluation of the plate was performed in the absorption reflection mode at 282 nm using Win-Cats software by considering dilution factor of sample.

### Research protocol approval

The experimental protocol was approved by the Institutional Animal Ethics Committee (IAEC) constituted in accordance with the rules and guidelines of the Committee for the Purpose of Control and Supervision on Experimental Animals (CPCSEA), India.

### Experimental animals

Male Wistar rats weighing between 200 and 230 g and Swiss albino mice (18–23 g) were purchased from the National Institute of Bioscience, Pune, India. The animals were housed at an ambient temperature of 25±2 °C, relative humidity 50±2% and light and dark cycle (12 h light/dark). The animals had access to pellet diet (Chakan oil mills, Pune) and water *ad libitum*.

### Acute oral toxicity study

Swiss albino mice of either sex were subjected to acute toxicity studies as per guideline (AOT No. 425) suggested by the Organization for Economic Co-operation and Development 2001. The mice were observed by housing them individually in polypropylene metabolic cages continuously for 2 h for behavioral, neurological and autonomic profiles and for any lethality during the following 48 h.

### Selection of FLC dose

The study was carried out using three doses of FLC, *i.e.* 125 mg/kg, 250 mg/kg and 500 mg/kg. The results of the lower two doses, *i.e.* 125 mg/kg and 250 mg/kg, are not presented as no significant activity was observed.

### Experimental design and protocol

A total of 18 animals were randomly divided into three groups comprising of six animals per group.

Group I: control group, animals received distilled water as a vehicle for 8 days and normal saline (s.c.) on day 9 and 10.

Groups II: animals received ISO (5.25 mg/kg, s.c. on day 9 and 8.5 mg/kg on day 10 in normal saline) (Mali & Bodhankar, 2010).

Groups III: received FLC (500 mg/kg, p.o.) in distilled water for 8 days and then ISO (5.25 mg/kg, s.c.) on day 9 and 8.5 mg/kg on day 10 in normal saline).

### Serum parameters

After 24 hr of the last dose of ISO, blood was collected from the retro-orbital plexus of each rat under mild ether anesthaesia (TKM Pharma, India) for determination of biochemical parameters. Serum was separated in cryocentrifuge (Eppendroff, India) at 4 °C at 6 000 rpm. for 15 min and lactate dehydrogenase (LDH), creatine phosphokinase-MB isoenzyme (CK-MB) and aspartate transaminase (AST) were measured by using standard kits according to the manufacturer's instruction manual (Merck Specialities Pvt. Ltd. India) using an autoanalyser (Nihon Kohden, Japan).

### Haemodynamic parameters

After 24 hr of the last dose of ISO, the animals were anaesthetised by urethane (1.25 g/kg) intrapritoneally. The right carotid artery of each rat was cannulated for the measurement of heart rate (HR), blood pressure (BP) systolic blood pressure (SBP), diastolic blood pressure (DBP) and mean aterial blood pressure (MABP). The cannula was filled with heparinised saline and connected to a pressure transducer. The haemodynamic parameters were recorded by eight-channel-power lab (AD Instruments, Australia) having LABCHART-6 Pro software. The percentage decrease in BP, SBP, DBP, MABP was calculated by the formula:

%=reading of treated group/reading of control group×100

After recording of haemodynamic parameters, the animals were euthanised and the heart was removed and placed in 10% formalin solution. The organ specimens were subjected to dehydration with xylene (one hour each) and alcohol of 70, 90 and 100% strength each for two hours. The infiltration and impregnation was carried out by treatment with paraffin wax twice for each time for one hour. Parrafin wax was used to prepare paraffin L molds. Specimens were cut into sections of 3–5 mm thickness and stained with haematoxylin and eosin (H & E). The sections were mounted by diestrene phthalate xylene (D.P.X.). The parameters of histopathology assessment of heart sections were inflammation, necrosis and congestion. The grading system used for assessment of parameters was [-: absence of change; +: 0–30% area shows changes;+ +: 30–60% area shows changes;+ + +: 60–100 area shows changes].

### Statistical analysis

Data were expressed as the mean±S.E.M. Statistical analysis was carried out by one-way ANOVA followed by *post hoc* Bonferroni test using graphpad prism 5.00 for Windows, GraphPad Software, San Diego California USA, www.graphpad.com. The *p-*value was considered significant when *p*<0.05.

## Results

### Chromatography

**Figure 1:**
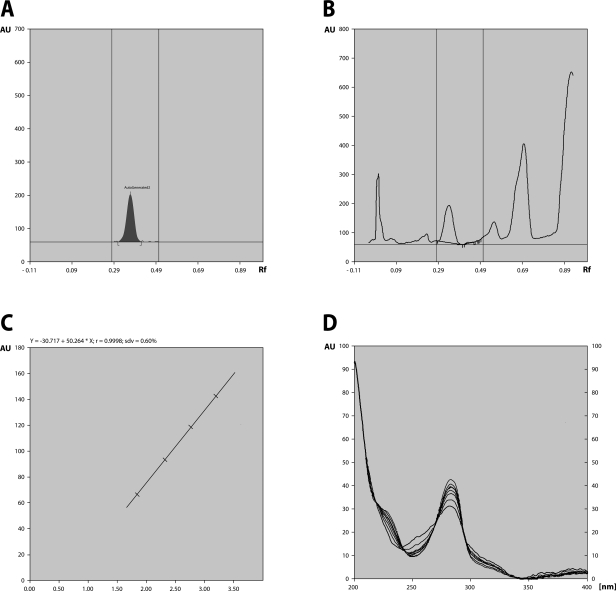
Chromatography. **A**: HPTLC densitogram of SDG standard; **B**: HPTLC densitogram of FLC; **C**: Calibration curves in linear in the range of 200, 400, 800, 1 600, 2 400, 3 200 ng for SDG standard; **D**: UV spectra of standard SDG and SDG present in the FLC samples obtained by HPTLC spot scanning from 200 to 400 nm.

With some modification in compositions of mobile phases used by Coran *et al*. (2004), we got better resolution of SDG. Peaks corresponding to SDG were recorded at Rf 0.35 (Figure 1A). FLC, when subjected to HPTLC, showed the presence of SDG peaks at the same Rf (Figure 1B). The calibration curves were linear in the range of 200, 400, 800, 1 600, 2 400, 3 200 ng for SDG (Figure 1C). Peak purity tests of SDG were also conducted by comparing UV visible spectra of SDG standard and sample track (Figure 1D). The SDG content in FLC was found to be 40 mg/g, from calibration curve Y= -30.717+50.254×X, r= 0.9998, Standard deviation (Sdv) =0.60 (Figure 1D).

### Acute toxicity studies

In acute oral toxicity studies, no changes in the behaviour and autonomic profiles and no mortality were observed in treated and control groups of the mice up to the dose of 5 000 mg/kg.

### Effect of FLC on serum parameters in rats after isoprenalin induced myocardial infarction

The effects of FLC on serum marker enzymes LDH, CK-MB and AST are shown in Table 1. ISO-treated group of animals showed significant (*p*<0.01) increase in the activity of CK-MB compared to the control group. Treatments with FLC 500 mg/kg to ISO challenged animals significantly decreased the activities of CK-MB elevated by ISO (*p*<0.01). LDH was significantly higher in ISO treated rats compared to control group (*p*<0.001) and FLC at the dose of 500 mg/kg decreased LDH, though not to control value. Isoprenalin increased significantly AST in ISO treated group compared to control group (*p*<0.05). FLC 500 mg/kg was effective in reducing AST compared to ISO group.

**Table 1:** Effect of FLC on serum parameters in rats after isoprenalin-induced myocardial infarction.

	Control	ISO	FLC 500 mg/kg
**CK-MB**	1211± 338.7	2703±139.2 ^a^	1286±301.9 ^b^
**LDH**	2161±487.5	8153±576.6 ^c^	5747±1354
**AST**	194.2±33.61	338.5±34.34 ^d^	271.2±31.56

Values are expressed as Mean ± SEM. Data was analyzed by One-way ANOVA followed by *post hoc* Bonferroni test. *p*<0.05 considered as significant.

a=**p<0.01 compared to control group

b=**p<0.01 compared to ISO group

c=***p<0.001 compared to control group

d=**p<0.05 compared to control group.

### Haemodynamic pattern

In the control-vehicle treated animals the recorded heart rate (HR) was 322.0±8.59. Isoprenalin significantly increased the heart rate (397.8±13.62) *p*<0.05 compared to of the control group. In FLC+ISO group the heart rate (364.1±27.83) did not increase significantly compared to control and was lesser than that of ISO group. The result thus indicated that FLC pretreatment for 8 days was effective in arresting isoprenalin-induced tachycardia (Table 2).

**Table 2:** Effect of isoprenalin on heart rate, blood pressure, diastolic pressure, systolic pressure and mean arterial blood pressure after 10-day treatment with FLC.

	Control	ISO	FLC 500 mg/kg
**HR**	322.0±8.595	397.8±13.62 ^a^	364.1±27.83
**BP**	109.7±4.673	100.9±3.666	94.82±3.048 ^b^
**SBP**	112.9±5.238	104.9±3.416	96.91±3.764 ^b^
**DBP**	105.4±4.117	95.7±4.548	91.90±2.967
**MABP**	107.9±4.445	98.7±3.968	93.57±2.933 ^b^

Values are expressed as Mean±SEM. Data were analyzed by one-way ANOVA followed by *post hoc* Bonferroni test using GraphPad Prism 5.00 for Windows, GraphPad Software, San Diego California USA. The p value was considered significant when *p*<0.05.

a=*p<0.05 compared to control group

b=*p<0.05 compared to ISO group.

### Effect of FLC and ISO on blood pressure

In the control animals treated with vehicle the recorded BP values were following: recorded BP 109±4.67 mmHg, systolic BP was 112.9±5.23 mmHg, diastolic BP was 105.4±4.11 mmHg and the mean arterial BP was 107.9±4.44 mmHg. The values obtained in the control animals treated with ISO were following: recorded BP was 100.9±3.66 mmHg (8%), systolic BP was 104.9±3.41 mmHg (7%), diastlic BP was 95.7±4.54 mmHg (12.8%) and mean arterial BP was 98.7±3.96 mmHg (8.5%) respectively. FLC+ISO (500 mg/kg) decreased BP to 94.82±3.04 mmHg (*p*<0.05) (13.5%), systolic BP to 96.91±3.76 mmHg (*p*<0.05) (14%), diastolic BP to 91.90±2.96 mmHg (12.8%) and mean arterial BP to 93.57±2.93 mmHg (*p*<0.05) (13%) compared to ISO alone (Table 2).

### Histopathology

**Figure 2:**
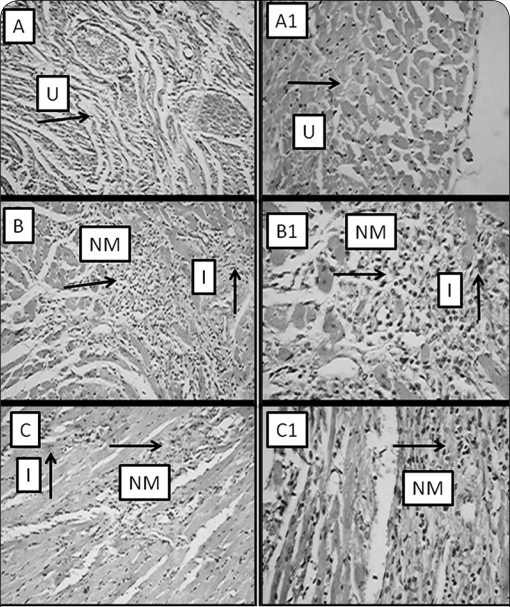
Photomicrographs of histopathological changes of rat heart at 20× and 40×. A (20×) (-, absence of change), A1 (40×) Control group, B (20×) (+ +, 30–60% necrosis), B1 (40×) ISO (8.5 mg/kg, s. c.) group, C (20×) (+, 0–30% necrosis), C1 (40×) FLC (500 mg/kg, p.o.)+ISO (8.5 mg/kg s. c.) U - Unremarkable, NM - Necrotic myocardial cells, I - Inflammatory cells.

Figure 2 illustrates the section of heart tissue in control rats (A), in rats that received ISO only (B) and in rat that received ISO+FLC (500 mg/kg) (C). The histopathology of the heart of the control group showed absence of inflammation, necrosis and congestion (graded as -: absence of changes). Isoprenalin administration in the control group showed inflammation (++), necrosis (++) and congestion (+) which means that a 30–60% area of the rat heart was inflamed, necrosis occurred in a 30–60% area of heart and 0–30% was congested. Inflammation and congestion in large areas of the heart confirmed cardiotoxicity in rats. On the other hand, inflammation, necrosis and congestion were observed in a smaller area of the heart (+, 0–30%) in FLC+ISO (500 mg/kg). The results of histopathological analysis thus indicated that the rat heart was partially protected by pre-treatment with FLC (500 mg/kg) against isoprenalin-induced cardiotoxicity.

## Discussion

Heart failure is a serious clinical syndrome with progressive myocardial dysfunction and a poor clinical outcome. Several studies have confirmed the role of inflammation and oxidative stress in progression of heart failure. Isoproterenol-induced myocardial necrosis is a well established model of myocardial infarction (MI) in rats (Rona, 1985). It has been established that catecholamines in large doses produce myocardial necrosis. Previously we have reported *in vitro* antioxidant activity of flaxseed (Zanwar *et al*., 2010) and *in vivo* antioxidant activity was reported by Rajesha *et al*. (2006). Therefore, the present study evaluated the role of FLC in fighting isoprenalin-associated damage in the myocardium of MI rats. Along with various proposed mechanisms of isoprenalin-induced MI, generation of free radicals is one of the important causative factors (Singal *et al*., 1983).

In the present investigation, the observed changes in haemodynamic parameters after administration of isoprenalin in control rats were comparable to earlier reported ones (Mali & Bodhankar, 2010). Isoprenalin injection on the 9^th^ and 10^th^ day significantly increased the HR compared to that of the control group. Increased myocardial oxygen demand leads to ischaemic necrosis of the myocardium in rats. ISO+FLC produced a non-significant decrease in HR compared to that of ISO. After isoprenalin treatment, 24-h blood pressure measurement showed a non-significant decrease in MABP. FLC pretreatment attenuated the hypotensive effect of isoprenalin and tachycardia. Isoprenalin produces relative ischaemia or hypoxia due to myocardial hyperactivity and coronary hypotension (Balazs & Ferranst, 1978) and induces myocardial ischaemia due to cytosolic Ca^2+^ overload (Rona, 1985). Additionally, isoprenalin causes myocardial ischaemia due to excessive production of free radicals resulting from oxidative metabolism of catecholamines (Singal *et al*., 1983).

Apart from haemodynamic parameters, several diagnostic marker enzymes like CK-MB isoenzyme and LDH present in the myocardium are used as predictor of pathological changes in the heart. These enzymes are released into the extracellular fluid during myocardial injury (Balazs & Ferranst, 1978). CK-MB is a standard marker of myocardial injury or death. CK-MB leaks out from the myocardium due to disintegration of the contractile apparatus and increased sarcoplasmic permeability (Mair *et al*., 1994). In the isoprenalin group the significant increase in CK-MB indicated myocyte injury. A lesser increase in CK-MB after isoprenalin in the FLC-treated group indicated cardioprotective activity of FLC. So our experimental data showed lowered activities of these enzymes in FLC-treated rats than in isoprenalin-treated rats, indicating protection against necrotic damage of the myocardial membrane. The lower LDH level in FLC-treated group, although is not significant, following isoprenalin insult, further supports the protective effect. Increase in the serum level of AST in the isoprenalin alone group compared to the control group indicates myocardial necrosis. FLC-treated rats had a lower level of AST, indicating cardioprotection. The activity of FLC also seems to preserve the structural and functional integrity of the myocardial membrane, as evident from the reduction in the elevated levels of these serum marker enzymes in rats pretreated with FLC when compared to the ISO alone group, establishing thus the cardioprotective effect of FLC.

Isoprenalin, a potent synthetic catecholamine, when administered to animals in high doses produces ‘‘infarct-like’’ lesions in the heart, which are similar to those found in acute myocardial infarction and sudden death in man (Baroldi, 1974). The pathogenesis of AMI has not yet been understood fully, despite studies on dose and rate of ISO-induced cardiotoxicity. ISO administration of 5.25 mg/kg on day 9 and 8.5 mg/kg on day 10 induced moderate lesions in the myocardium and significantly altered various biochemical parameters. The cardioprotective activity of FLC was evaluated against these doses. Isoprenalin produces relative ischaemia or hypoxia due to myocardial hyperactivity and coronary hypotension, and induces myocardial ischaemia due to cytosolic Ca^2+^ overload. Additionally, isoprenalin causes myocardial ischaemia due to excessive production of free radicals resulting from oxidative metabolism of catecholamines (Rona, 1985). Although cardiotoxicity could occur primarily via adrenoceptor activation, there is increasing evidence that it may also occur through oxidative mechanisms. Dhalla *et al*. (2000) reported that excess catecholamines affect Ca^2+^-transport mechanisms primarily via oxidation reactions involving free-radical-mediated damage and antioxidants may be beneficial in stress-induced heart disease. A considerable amount of biochemical, physiological and pharmacological data support the link between free radicals and cardiovascular tissue injury, associated mainly with increase in vascular ROS production. Our earlier studies were indicative of the antioxidant property of FLC as a scavenger of different free radicals, including anion superoxide (O_2_^-^), hydroxyl radicals (OH^•^) and hydroxyl observed in several biological models (Zanwar *et al*., 2010). FLC might reduce or prevent excessive production of free radicals, exhibiting its cardioprotective effect. Histopathological finding of the FLC (500 mg/kg, p.o.) pre-treated myocardium showed reduction in necrosis and inflammation seen with isoproterenol treatment. FLC (500 mg/kg) treated normal rats showed only 0–30% cardiac necrotic architecture. These data further confirmed the cardioprotective action of oral administration of FLC. FLC, which contains SDG lignan, is biphenolic and an effective scavenger of reactive oxygen species and can inhibit lipid peroxidation through chelation of transition metal ions or their chain-breaking antioxidant activity. The observed effects might be due to the antioxidant effect of SDG present in FLC, redirecting the haemodynamic, biochemical, and histopathological changes towards normal values.

## Conclusion

It is concluded that 10-day administration of FLC reduced isoprenalin-induced tachycardia. Protection from cardiotoxic effect of isoprenalin in FLC-pretreated animals was established by haemodynamic, biochemical, and histopathological results. The antioxidant effect appears to contribute to the cardioprotective effect of flax lignan concentrate in isoprenalin-induced cardiotoxicity.
